# Limited adaptability of virtual memory CD8 T cells to chronic viral infection

**DOI:** 10.3389/fimmu.2026.1804320

**Published:** 2026-05-15

**Authors:** Yamato Sajiki, Yi-Chung Huang, Charles M. Perkins, Satomi Ando, Koichi Araki

**Affiliations:** 1 Division of Infectious Diseases, Center for Inflammation and Tolerance, Cincinnati Children’s Hospital Medical Center, Cincinnati, OH, United States; 2 Immunology Graduate Program, University of Cincinnati College of Medicine, Cincinnati, OH, United States; 3 Department of Pediatrics, University of Cincinnati College of Medicine, Cincinnati, OH, United States

**Keywords:** CD8 T cells, chronic infection, immunotherapy, t cell exhaustion, virtual memory

## Abstract

Antigen-inexperienced CD8 T cells include naïve and virtual memory (VM) subsets. VM CD8 T cells exhibit a memory-like phenotype despite lacking prior exposure to their specific antigen. While they can efficiently respond to and control acute infections where pathogens are cleared, their response during chronic infection remains poorly characterized. Using a chronic lymphocytic choriomeningitis virus infection model, we found that VM CD8 T cells exhibit a diminished response to persistent antigen stimulation compared to naïve CD8 T cells. Mechanistically, VM CD8 T cells show impaired engagement of the T cell exhaustion program due to lower TOX expression, resulting in a marked reduction of TCF1^+^ stem-like CD8 T cells which are critical for sustaining antigen-specific responses during chronic infection. Instead, VM CD8 T cells preferentially differentiate into KLRG1^+^PD-1^-^ cells, a population rarely observed in naïve-derived progeny. Moreover, VM-derived CD8 T cells exhibit limited expansion following PD-1 blockade, consistent with their reduced TCF1^+^ stem-like compartment. In summary, VM CD8 T cells fail to properly engage the exhaustion program during chronic viral infection, leading to a fundamental limitation in their adaptability to persistent antigen-stimulation.

## 1 Introduction

Among the pool of antigen-inexperienced CD8 T cells, there are two subsets: naïve and virtual memory (VM) CD8 T cells. VM CD8 T cells are antigen-inexperienced but phenotypically distinct from naïve CD8 T cells (1–3). While lacking prior exposure to their specific antigen, VM CD8 T cells exhibit memory-like phenotypes, and their development is antigen-independent and instead regulated by cytokine-driven mechanisms (1, 4–7). The phenotype of VM CD8 T cells has been characterized as CD44^Hi^CD49d^Lo^CD122^Hi^CD62L^Hi^, which distinguish them from naïve (CD44^Lo^CD49d^Lo^CD122^Lo^CD62L^Hi^) and true memory (CD44^Hi^CD49d^Hi^CD122^Hi^CD62L^Lo/Hi^) CD8 T cells in mice (2, 8). To date, studies of VM CD8 T cells have largely focused on their functions in antigen-independent contexts (9). One previous study has demonstrated that, similar to true memory CD8 T cells, antigen-specific VM CD8 T cells can efficiently respond to and control an acute *Listeria monocytogenes* infection (10). In contrast, how antigen-specific VM CD8 T cells function under chronic antigen exposure remains poorly defined.

During chronic infection and cancer, CD8 T cells undergo persistent antigen stimulation, leading to a progressive loss of effector function. This dysfunctional state, known as T cell exhaustion, is characterized by sustained expression of inhibitory receptors, reduced proliferative capacity, and diminished cytokine production and cytotoxic activity (11–13). During T cell exhaustion, multiple subsets emerge including stem-like (PD-1^+^TCF1^+^TIM3^–^), transitory (PD-1^+^TCF1^–^TIM3^+^CX3CR1^+^), and terminally exhausted (PD-1^+^TCF1^–^TIM3^+^CD101^+^) CD8 T cells (14, 15). Among these subsets, TCF1^+^ stem-like CD8 T cells play a crucial role in maintaining the antigen-specific CD8 T cell pool during chronic infection and cancer by self-renewal and generating the two differentiated TIM3^+^ subsets (16–19). Furthermore, these cells can proliferate in response to PD-1 targeted immune therapies and give rise to the transitory subset which retains effector function (18). Previous studies have identified the transcriptional factor TOX as a key regulator of T cell exhaustion (20–22). TOX-deficient CD8 T cells failed to generate stem-like CD8 T cells and instead preferentially differentiated into KLRG1^+^PD-1^–^ cells, a terminal effector subset typically formed during acute infection but rarely observed in chronic infection, ultimately leading to the loss of antigen-specific CD8 T cells. However, because these prior studies have focused on exhaustion arising from naïve CD8 T cells, the relationship between VM CD8 T cells and the differentiation of exhausted CD8 T cells during chronic infection and cancer remains to be elucidated.

In this study, we examined the response of VM CD8 T cells under persistent antigen-stimulation utilizing a chronic lymphocytic choriomeningitis virus (LCMV) clone-13 infection model. Specifically, we compared the responses of naive and VM CD8 T cells to LCMV clone-13 infection using an adoptive co-transfer approach. Furthermore, we investigated how progeny derived from these two populations respond to anti-PD-L1 antibody treatment.

## 2 Materials and methods

### 2.1 Mice

C57BL/6J (B6) mice (6- to 8-week-old, strain #: 000664) were purchased from The Jackson Laboratory. To generate CD45.1^+^CD45.2^+^ B6 mice, male CD45.1 B6 mice (strain #: 033076) were purchased from The Jackson Laboratory and bred with female CD45.2 B6 mice. TCR transgenic P14 mice were bred and maintained in our animal colony. All mice were housed under specific pathogen-free conditions and all experiments were performed under the Institutional Animal Care and Use Committee of Cincinnati Children’s Hospital approved protocols (protocol # 2023-1039).

### 2.2 Viral infection

For acute infection, mice were intraperitoneally infected with LCMV Armstrong (2 × 10^5^ pfu/mouse). For chronic infection, mice were intravenously infected with LCMV clone-13 (2 × 10^6^ pfu/mouse). In chronic infection experiments, CD4 T cell depletion was performed by intraperitoneal administration of 300 μg of anti-CD4 antibody (clone: GK1.5; Leinco Technologies) on the day of infection and one day post-infection, unless otherwise indicated.

### 2.3 Lymphocyte isolation

Blood samples were collected by submandibular bleeding, and peripheral blood mononuclear cells (PBMCs) were isolated using Lymphocyte Separation Medium (Corning). Following the isolation of PBMCs, red blood cells were lysed using Red Blood Cell Lysing Buffer Hybri-Max (Sigma-Aldrich). For splenocyte isolation, the spleen was harvested from mice and splenic cells were released by mechanical disruption and treated with Red Blood Cell Lysing Buffer Hybri-Max. The cells were then washed with RPMI 1640 (Gibco) containing 2% fetal bovine serum (FBS; Gibco).

### 2.4 Flow cytometry

For surface staining, cells were stained with an antibody cocktail for 30 minutes on ice and fixed by the Cytofix/Cytoperm kit (BD Biosciences). Following fixation, intracellular staining for granzyme B was performed. Intranuclear staining of transcriptional factors, TCF1 and TOX, was performed after cell permeabilization using the Foxp3/transcription factor staining buffer set (eBioscience). The following antibodies were purchased from BioLegend: CD8 (clone: 53-6.7), CD45.2 (clone: 104), CD45.1 (clone: A20), KLRG1 (clone: 2F1/KLRG1), PD-1 (clone: RMP1–30 or 29F.1A12), TIM3 (clone: RMT3-23), CD49d (clone: R1-2), CD122 (clone: 5H4), and CD44 (clone: IM7). The following antibodies were purchased from eBioscience: TOX (clone: TXRX10) and CD62L (clone: MEL-14). An anti-TCF1 antibody (clone: S33-966) was purchased from BD Biosciences. An anti-TIM3 antibody (clone: 215008) was purchased from R&D Systems. An anti-CD44 antibody (clone: REA664) was purchased from Miltenyi Biotec. An anti-granzyme B antibody (clone: GB12) was purchased from Invitrogen. Live cells were determined using the LIVE/DEAD Fixable Near-IR Dead Cell Stain Kit (Invitrogen). Stained samples were acquired using BD FACSCanto (BD Biosciences), LSRFortessa (BD Biosciences), or Cytek Aurora (Cytek Biosciences) and analyzed using FlowJo software (BD Biosciences).

### 2.5 Adoptive transfer

For the analysis of CD44^Lo^ and CD44^Hi^ CD8 T cell responses during acute and chronic infections, CD44^Lo^ and CD44^Hi^ P14 TCR transgenic CD8 T cells were sorted from the spleen of TCR transgenic P14 mice using FACSymphony S6 (BD Biosciences). The Fc-mutated anti-CD44 antibody (clone: REA664), which lacks Fc receptor binding ability, was used for cell sorting to prevent anti-CD44 antibody-mediated depletion of sorted cells after adoptive transfer. Sorted CD44^Hi^ P14 cells (1 × 10^3^ cells/mouse, either CD45.1^+/+^ or CD45.1^+^/CD45.2^+^) were adoptively co-transferred with an equal number of congenically distinct sorted CD44^Lo^ P14 cells into recipient B6 mice (CD45.2^+/+^). On the following day, the recipient mice were infected with either LCMV Armstrong or clone-13. To control potential congenic−dependent effects, reciprocal congenic labeling was performed by co−transferring CD44^Lo^ and CD44^Hi^ P14 CD8 T cells using two CD45 configurations (CD44^Lo^ CD45.1^+/+^ with CD44^Hi^ CD45.1^+^/CD45.2^+^ and vice versa). Both reciprocal labeling strategies yielded comparable results with no detectable congenic−dependent differences. For the analysis of non-transgenic polyclonal CD44^Lo^ and CD44^Hi^ CD8 T cell responses, CD44^Lo^ and CD44^Hi^ CD8 T cells were sorted from the spleen of naïve B6 mice using FACSymphony S6. Sorted CD8 T cells (3 × 10^5^ cells/mouse) were separately transferred into congenically marked recipient B6 mice. On the following day, the recipient mice were infected with LCMV clone-13.

### 2.6 Anti-PD-L1 antibody treatment

On day >30 post-LCMV clone-13 infection, mice were treated with 200 μg of anti-PD-L1 antibody (clone: 10F.9G2, Leinco Technologies) every 3 days for a total of 5 doses. A day after final dose, the spleen was harvested from the mice to examine the responses of CD44^Lo^- and CD44^Hi^-derived P14 populations following PD-1 blockade.

### 2.7 Tetramer enrichment

For B6 mice prior to infection, splenic single cell suspensions were incubated with FcR blocker (STEMCELL Technologies) and tetramers (APC-conjugated DbGP33, PE-conjugated DbGP33, APC-conjugated DbGP276, and PE-conjugated DbGP276) for 30 min on ice. Dual tetramer staining enhances the specificity of the assay (23). Cells were then washed once with MACS buffer (PBS containing 2% FBS and 2 mM EDTA (Invitrogen)) and the tetramer positive cells were pulled down using EasySep Mouse APC Positive Selection Kit II (STEMCELL Technologies) according to the manufacturer’s instructions. For the analysis of LCMV-specific CD8 T cells derived from donor CD44^Lo^ and CD44^Hi^ CD8 T cells from B6 mice (non-P14) during LCMV clone-13 infection, tetramer enrichment with amplification of APC signaling was performed. Splenic single cell suspensions were incubated with an anti-CD16/CD32 antibody (BioLegend) for 15 minutes on ice to block Fc receptors and were stained with APC-conjugated DbGP33 and DbGP276 tetramers for 30 min on ice. Cells were then washed twice with MACS buffer and were mixed with Biotin anti-APC Antibody (clone: APC003, BioLegend). After incubation for 15 minutes on ice, cells were washed twice with MACS buffer and mixed with Streptavidin, Allophycocyanin, crosslinked, conjugate (Invitrogen). After incubation for 15 minutes on ice, cells were washed twice with MACS buffer and incubated with anti-APC microbeads (Miltenyi Biotec) for 15 minutes at 4 °C. After washing the cells twice with MACS buffer, the tetramer positive cells were pulled down using LS-columns (Miltenyi Biotec) according to the manufacturer’s instructions.

### 2.8 Statistical analysis

Statistical significance was evaluated using the unpaired *t* test, paired *t* test, multiple paired *t* test, or one-way ANOVA with Tukey’s multiple comparison test. Log-transformed values were used for statistical comparisons in cell numbers. Statistical analyses were performed with Prism 10 (GraphPad).

## 3 Results

### 3.1 Virtual memory CD8 T cells exhibit a reduced response to chronic infection

**Figure 1:**
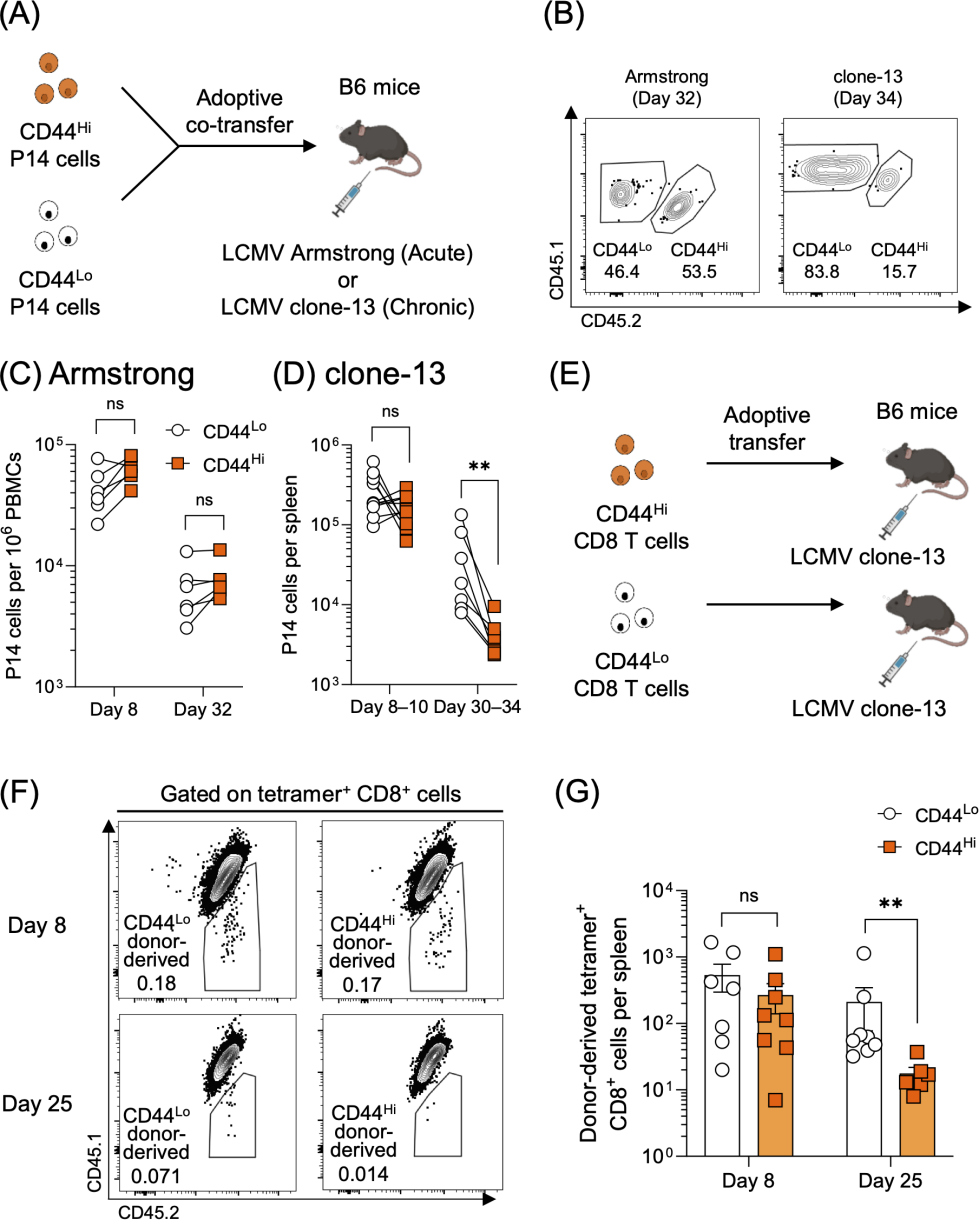
VM CD8 T cells exhibit a diminished response during chronic infection. **(A)** Experimental design for panels **(B–D)**. Congenically marked CD44^Lo^ and CD44^Hi^ P14 CD8 T cells (1,000 cells each) were adoptively co-transferred into B6 mice, followed by LCMV Armstrong or clone-13 infection. **(B)** Representative flow plots were gated on P14 CD8 T cells on day 32 post-LCMV Armstrong or day 34 post-LCMV clone-13 infection. Gating strategy is shown in **Supplementary Figure 2A**. **(C, D)** Numbers of CD44^Lo^- and CD44^Hi^-derived P14 CD8 T cells in blood on days 8 and 32 post-LCMV Armstrong infection **(C)** and in the spleens on days 8–10 and 30–34 post-LCMV clone-13 infection **(D)**. PBMC data on days 8 and 32 post-LCMV Armstrong infection were obtained from same mice. Data are pooled from 2 independent experiments with 6 or more mice per group. Each symbol represents an individual mouse, and lines indicate paired comparisons within the same mice. **(E)** Experimental design for panels **(F, G)**. CD44^Lo^ and CD44^Hi^ CD8 T cells (3 × 10^5^ cells/mouse), sorted from the spleen of B6 mice, were separately transferred into congenically distinct B6 mice, followed by LCMV clone-13 infection. These mice were not treated with the anti-CD4 antibody. On days 8 and 25 post-infection, the spleens were harvested from the infected mice, and tetramer enrichment was performed to examine the response of donor-derived GP33- and GP276-specific CD8 T cells. **(F)** Representative plots were gated on DbGP33^+^ and DbGP276^+^ CD8 T cells. Gating strategy is shown in **Supplementary Figure 2B**. **(G)** The number of donor-derived DbGP33^+^ and DbGP276^+^ CD8 T cells in the spleens on days 8 and 25 post-LCMV clone-13 infection. Data are pooled from 2 independent experiments with 6 or more mice per group. Each symbol represents an individual mouse, bars indicate the mean, and error bars indicate SEM. Statistical analyses were performed using multiple paired **(C, D)** or multiple unpaired **(G)** *t* test. ns, not significant; ** *p* < 0.01.

To investigate whether and how the response of VM CD8 T cells to infection differs from that of naïve CD8 T cells, we used P14 TCR transgenic CD8 T cells which express a TCR specific for the LCMV GP33 epitope. A previous study showed that P14 CD8 T cells contain a CD44^Hi^ population (24), and we confirmed that these CD44^Hi^ P14 cells possessed a canonical virtual memory CD8 T cell phenotype, characterized by CD49d^Lo^CD122^Hi^CD62L^Hi^ expression (**Supplementary Figures 1A–C**). To examine the response of VM CD8 T cells, we sorted CD44^Lo^ and CD44^Hi^ P14 cells from the spleens of congenically distinct P14 TCR transgenic mice and adoptively co-transferred an equal number of the sorted two populations into recipient B6 mice, followed by either acute LCMV Armstrong or chronic LCMV clone-13 infection (**Figure 1A**, **Supplementary Figure 2A**). During LCMV Armstrong infection, both CD44^Lo^ and CD44^Hi^ P14 cells expanded and contracted similarly (**Figures 1B, C**). In contrast, the response of CD44^Hi^ P14 cells to chronic infection was markedly different from that of CD44^Lo^ P14 cells. Although similar numbers of CD8 T cells derived from CD44^Lo^ and CD44^Hi^ P14 cells were detected on days 8–10 post LCMV clone-13 infection, CD44^Hi^-derived P14 cells were significantly reduced compared to CD44^Lo^-derived P14 cells by days 30–34 post LCMV clone-13 infection (**Figures 1B, D**). These observations indicate that VM CD8 T cells are more detrimentally impacted by persistent antigen stimulation than naïve CD8 T cells. To confirm that our observations were not limited to TCR transgenic CD8 T cells, we next investigated the responses of CD44^Lo^ and CD44^Hi^ cells within non-transgenic polyclonal antigen-specific CD8 T cells. Using tetramer enrichment, we verified that non-transgenic LCMV-specific CD44^Hi^ CD8 T cells in the spleen of uninfected B6 mice showed a CD49d^Lo^ VM phenotype (**Supplementary Figure 3**), supporting the use of bulk CD44^Hi^ CD8 T cells as a source of LCMV-specific VM cells for adoptive transfer. CD44^Lo^ and CD44^Hi^ CD8 T cells were sorted from the spleen of B6 mice and separately transferred into congenically distinct recipients, followed by LCMV clone-13 infection (**Figure 1E**, **Supplementary Figure 2B**). Similar to the P14 data, while the numbers of LCMV-specific CD8 T cells were comparable between the two groups on day 8 post-infection, the CD44^Hi^ group exhibited significantly fewer cells than the CD44^Lo^ group at day 25 post-infection (**Figures 1F, G**). Taken together, these findings suggest that VM CD8 T cells cannot maintain their population upon persistent antigen-stimulation.

### 3.2 TCF1^+^ stem-like CD8 T cells are less abundant in the progeny derived from VM cells during chronic infection

**Figure 2:**
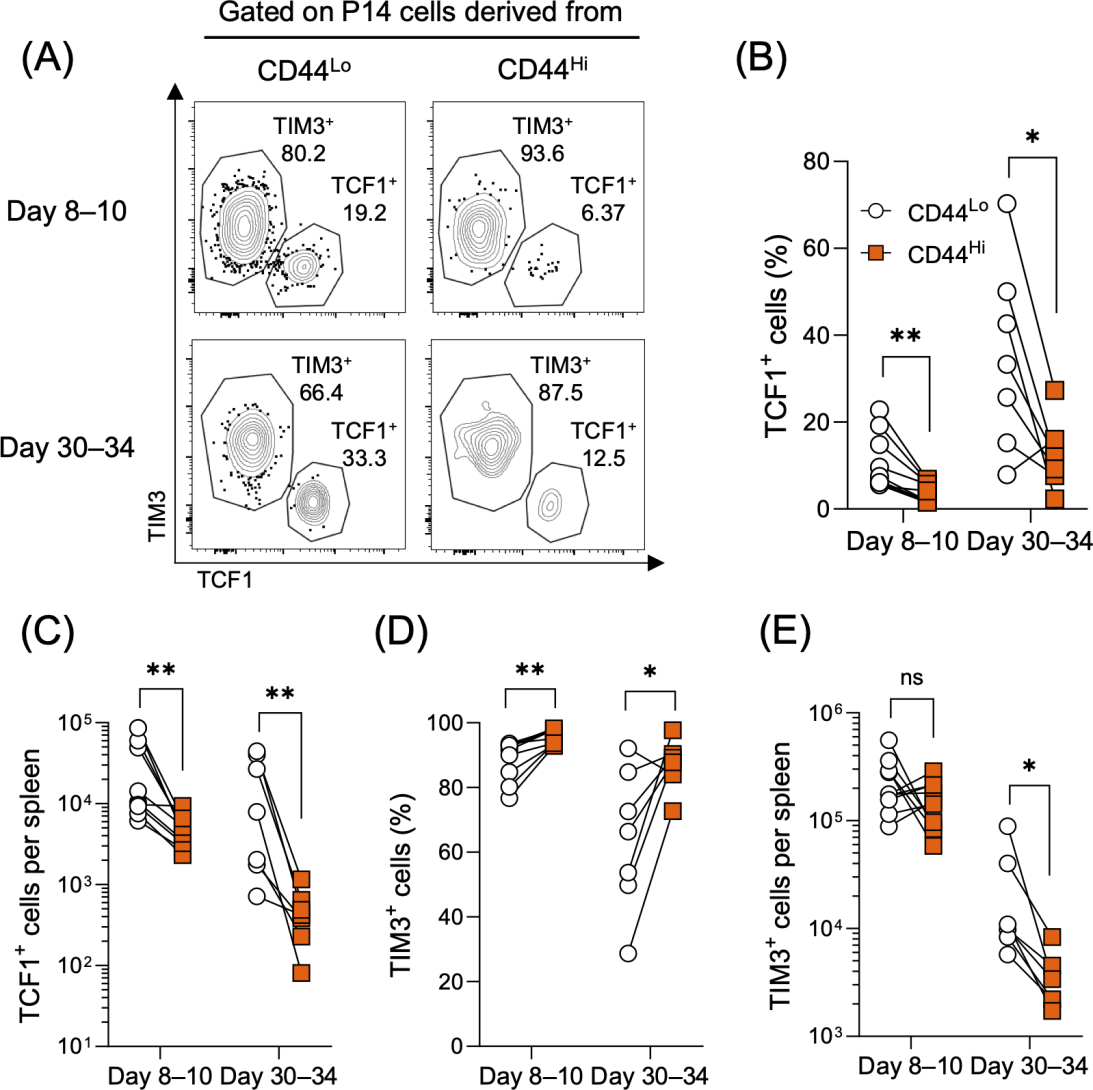
VM-derived CD8 T cells possess fewer TCF1^+^ stem-like cells during chronic infection. Experimental design is shown in **Figure 1A**. **(A)** Representative flow plots were gated on P14 CD8 T cells derived from either CD44^Lo^ or CD44^Hi^ cells on days 8–10 and 30–34 post-LCMV clone-13 infection. Gating strategy is shown in **Supplementary Figure 2A**. **(B–E)** Percentages of TCF1^+^ and TIM3^+^ progeny derived from CD44^Lo^ and CD44^Hi^ P14 CD8 T cells **(B, D)** and their absolute numbers **(C, E)** in the spleens on days 8–10 and 30–34 post-LCMV clone-13 infection. Data are pooled from 2 independent experiments with 6 or more mice per group. Each symbol represents an individual mouse, and lines indicate paired comparisons within the same mice. Statistical analyses were performed using multiple paired *t* test. ns, not significant; * *p* < 0.05; ** *p* < 0.01.

In the present study, we observed a significant reduction in VM-derived antigen-specific CD8 T cells during the contraction phase following chronic LCMV clone-13 infection. During chronic infection, the maintenance of antigen-specific CD8 T cells relies on TCF1^+^TIM3^–^ stem-like CD8 T cells which sustain the population through self-renewal and by generating differentiated TCF1^–^TIM3^+^ cells (17–19). Based on this, we hypothesized that the diminished response of VM CD8 T cells to chronic infection might result from a lower number of TCF1^+^ stem-like CD8 T cells. To test this hypothesis, we analyzed the formation of TCF1^+^TIM3^–^ and TCF1^–^TIM3^+^ subsets among the progeny of CD44^Lo^ and CD44^Hi^ CD8 T cell populations after LCMV clone-13 infection. Both frequencies and absolute numbers of TCF1^+^TIM3^–^ CD8 T cells were already significantly lower in CD44^Hi^-derived P14 cells on days 8–10 post-infection compared to CD44^Lo^-derived P14 cells and this difference remained through days 30–34 post-infection (**Figures 2A–C**). Due to the reduction of TCF1^+^TIM3^–^ CD8 T cells in CD44^Hi^-derived P14 cells early after infection, the frequency of TCF1^–^TIM3^+^ cells was significantly higher in this group compared to their CD44^Lo^-derived counterparts (**Figures 2A, D**, day 8–10), while their absolute numbers were comparable between the two groups (**Figure 2E**). By days 30–34 post-infection, the number of TCF1^–^TIM3^+^ cells in the CD44^Hi^ group was significantly reduced (**Figure 2E**). Although the experiments in **Figure 2** were performed in the absence of CD4 T cell help, impaired generation of TCF1^+^ stem-like cells from CD44^Hi^-derived CD8 T cells was also observed in the presence of CD4 T cells (**Supplementary Figures 4A–E**). Collectively, these findings demonstrate a substantial reduction in TCF1^+^ stem-like CD8 T cells in the progeny of CD44^Hi^ CD8 T cells, which likely contributes to the significant loss of the virus-specific CD8 T cell population during the contraction phase.

### 3.3 VM CD8 T cells fail to engage T cell exhaustion program due to lower TOX expression, leading to effector-biased differentiation

**Figure 3:**
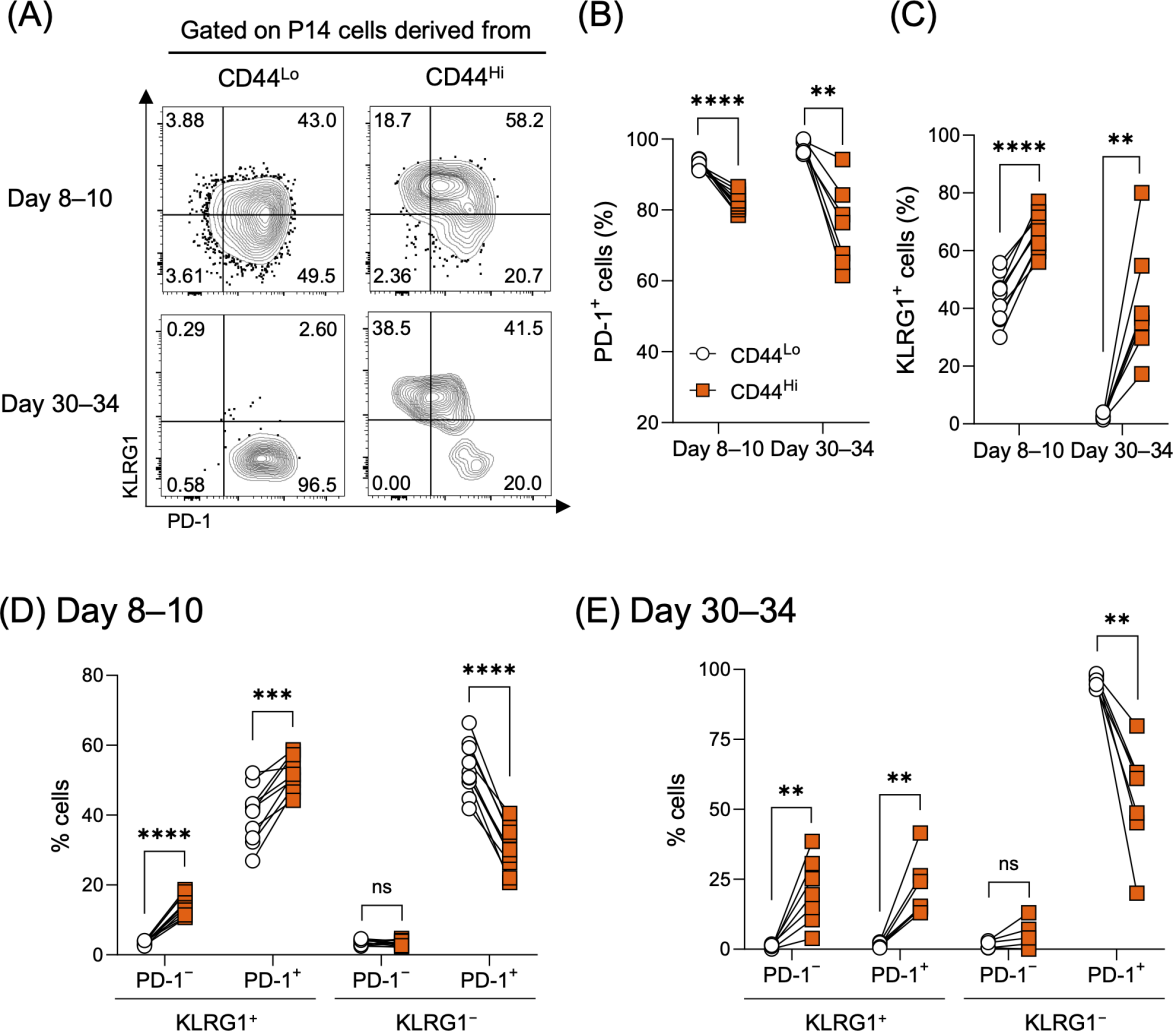
VM CD8 T cells exhibit a differentiation bias toward a unique phenotype during chronic infection. Experimental design is shown in **Figure 1A**. **(A)** Representative flow plots were gated on P14 CD8 T cells derived from either CD44^Lo^ or CD44^Hi^ cells on days 8–10 and 30–34 post-LCMV clone-13 infection. Gating strategy is shown in **Supplementary Figure 2A**. PD-1 and KLRG1 gates were defined as indicated in **Supplementary Figure 5A**. **(B, C)** Percentages of PD-1^+^ **(B)** and KLRG1^+^ **(C)** progeny derived from CD44^Lo^ and CD44^Hi^ P14 CD8 T cells in the spleens on days 8–10 and 30–34 post-LCMV clone-13 infection. **(D, E)** Percentages of four subsets (KLRG1^+^PD-1^–^, KLRG1^+^PD-1^+^, KLRG1^–^PD-1^–^, and KLRG1^–^PD-1^+^) in progeny derived from CD44^Lo^ and CD44^Hi^ P14 CD8 T cells in the spleens on days 8–10 **(D)** and 30–34 **(E)** post-LCMV clone-13 infection. Data are pooled from 2 independent experiments with 6 or more mice per group. Each symbol represents an individual mouse, and lines indicate paired comparisons within the same mice. Statistical analyses were performed using multiple paired *t* test. ns, not significant; ** *p* < 0.01; *** *p* < 0.001; **** *p* < 0.0001.

During chronic infection, CD8 T cells are committed to the T cell exhaustion program due to persistent antigen-stimulation, resulting in their differentiation into PD-1^+^ exhausted CD8 T cells (11–13). Consistent with this paradigm, the majority of cells derived from CD44^Lo^ P14 cells expressed PD-1 during both the early and late phases of LCMV clone-13 infection (**Figures 3A, B**, **Supplementary Figure 5A**). In sharp contrast, the frequency of PD-1^+^ cells in the CD44^Hi^-derived population was significantly lower compared to the CD44^Lo^ counterparts (**Figures 3A, B**). Interestingly, KLRG1 expression was significantly upregulated in cells derived from CD44^Hi^ P14 cells (**Figures 3A, C**). We next examined the relationship between KLRG1 and PD-1 expression. During the early phase of infection, KLRG1 expression was largely restricted to PD-1^+^ cells in the CD44^Lo^ P14-derived population, whereas, in the CD44^Hi^ P14-derived population, a substantial fraction of PD-1^–^ cells also expressed KLRG1 (**Figures 3A, D**). During the chronic phase, most CD44^Lo^−derived P14 cells were KLRG1^–^, whereas KLRG1-expressing cells derived from CD44^Hi^ P14 cells were still present in both PD-1^+^ and PD-1^–^ compartments (**Figures 3A, E**). Given that the KLRG1^+^PD-1^–^ subset is typically observed during acute infection as a terminal effector population (25, 26), we next examined granzyme B expression, another key effector molecule. Consistent with the KLRG1 and PD-1 expression profiles, granzyme B expression was significantly upregulated in the VM-derived population (**Supplementary Figures 5B–D**). Together, these data suggest that VM CD8 T cells fail to fully engage the T cell exhaustion program during chronic infection and are instead skewed toward effector differentiation.

**Figure 4:**
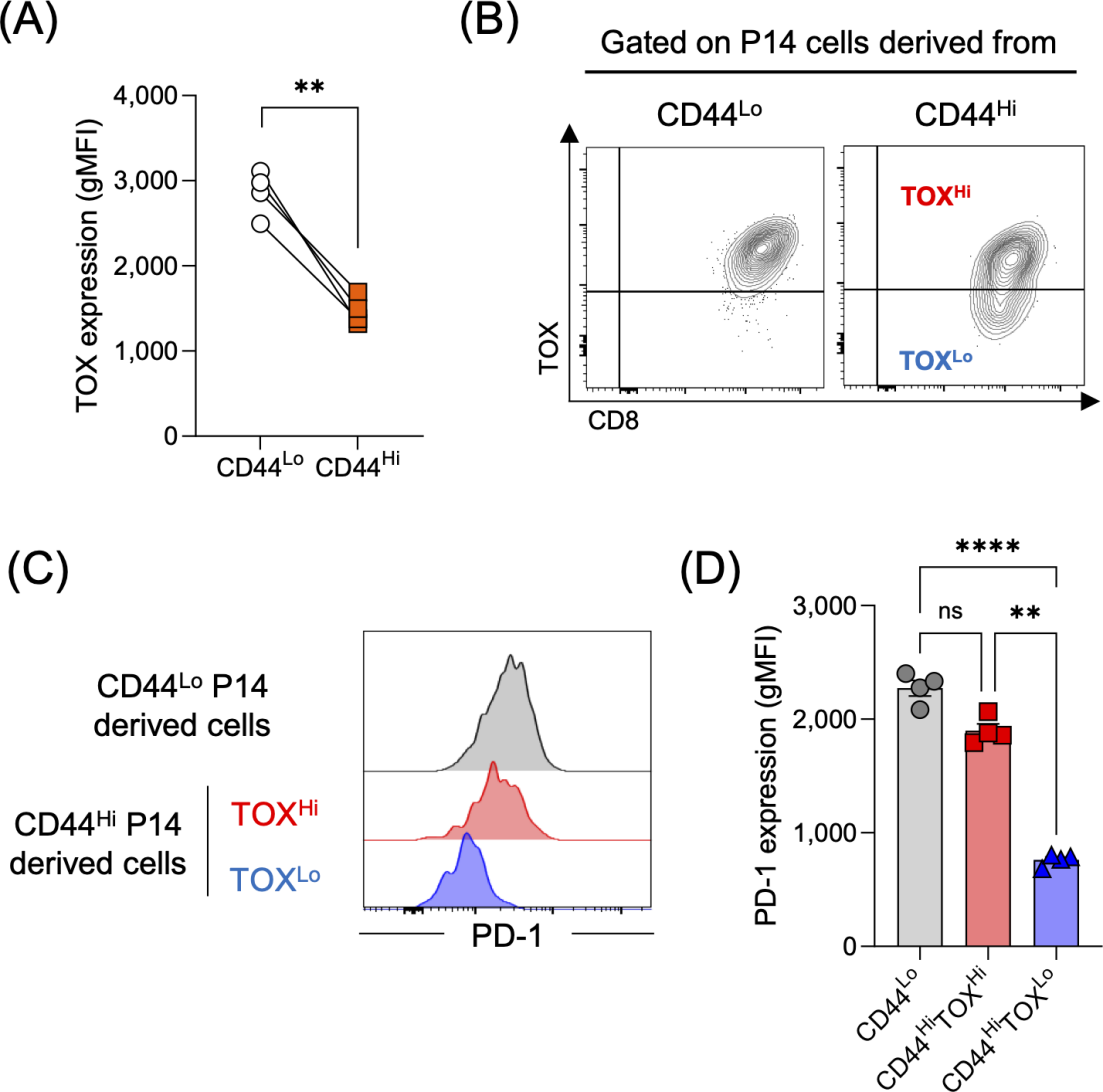
TOX expression is downregulated in VM-derived CD8 T cells during chronic infection. Experimental design is shown in **Figure 1A**. **(A, B)** The graph **(A)** indicates TOX expression in CD44^Lo^- and CD44^Hi^-derived P14 populations on day 10 post-LCMV clone-13 infection. Representative flow plots **(B)** were gated on P14 CD8 T cells derived from either CD44^Lo^ or CD44^Hi^ P14 CD8 T cells. Gating strategy is shown in **Supplementary Figure 2A**. TOX positive gates were defined as indicated in **Supplementary Figure 6**. Data are representative of 3 independent experiments with 4 mice per group. Each symbol represents an individual mouse, and lines indicate paired comparisons within the same mice. **(C, D)** Representative flow plots **(C)** were gated on either total P14 cells derived from CD44^Lo^ cells, TOX^Hi^ or TOX^Lo^ P14 cells derived from CD44^Hi^ cells. The graph **(D)** indicates PD-1 expression among these three populations on day 10 post-LCMV clone-13 infection. Data are representative of 3 independent experiments with 4 mice per group. Each symbol represents an individual mouse, bars indicate the mean, and error bars indicate SEM. Statistical analyses were performed using paired *t* test **(A)** or one-way ANOVA **(D)**. ns, not significant; ** *p* < 0.01; **** *p* < 0.0001.

Previous studies have shown that TOX transcriptionally and epigenetically regulates the T cell exhaustion program (20–22). Because the genetic or pharmacological disruption of the TOX-driven exhaustion program is known to prevent PD-1 upregulation and concurrently drive KLRG1 expression, we hypothesized that the unique KLRG1^+^PD-1^-^ phenotype observed in VM-derived progeny was directly linked to an intrinsic failure to sustain TOX expression. To test this hypothesis, we analyzed TOX expression in CD8 T cells derived from CD44^Lo^ and CD44^Hi^ P14 cells on day 10 post-LCMV clone-13 infection. TOX expression was significantly lower in the CD44^Hi^ group compared to the CD44^Lo^ group (**Figure 4A**). Most CD44^Lo^-derived P14 cells uniformly expressed TOX whereas the CD44^Hi^-derived population included a distinct TOX^Lo^ population (**Figure 4B**, **Supplementary Figure 6**). To determine whether reduced TOX expression is associated with impairment of the exhaustion program, we compared PD-1 expression among TOX^Hi^ and TOX^Lo^ cells within CD44^Hi^-derived population as well as in total CD44^Lo^-derived cells. As expected, PD-1 expression was substantially lower in the TOX^Lo^ cells compared to the other two groups (**Figures 4C, D**, **Supplementary Figure 6**). Collectively, these findings suggest that VM CD8 T cells exhibit impaired engagement of the T cell exhaustion program during chronic infection, likely due to reduced TOX expression.

### 3.4 VM-derived CD8 T cells undergo limited expansion in response to PD-1 blockade

**Figure 5:**
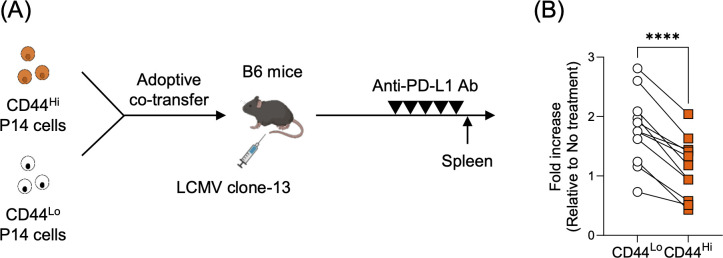
VM-derived CD8 T cells have limited capacity for expansion following PD-1 blockade. **(A)** Experimental design. Congenically marked CD44^Lo^ and CD44^Hi^ P14 CD8 T cells (1,000 cells each) were adoptively co-transferred into B6 mice, followed by LCMV clone-13 infection. During the chronic phase (day >30 post-infection), the mice were treated with anti-PD-L1 antibody (200 μg/mouse) every 3 days for a total of 5 doses. **(B)** Fold increase in CD44^Lo^− and CD44^Hi^−derived P14 cell numbers following anti-PD−L1 treatment, calculated relative to the geometric mean of untreated controls for each subset. Data are pooled from 2 independent experiments with 11 mice per group. Each symbol represents an individual mouse, and lines indicate paired comparisons within the same mice. Statistical analysis was performed using paired *t* test. **** *p* < 0.0001.

Previous studies have demonstrated that TCF1^+^ stem-like CD8 T cells are critical for the efficacy of PD-1/PD-L1 targeted immunotherapies, as PD-1 blockade acts on this population and induces a proliferative burst (15, 18). In this study, we showed that the number of TCF1^+^ stem-like CD8 T cells was significantly reduced in the VM CD8 T cell-derived population during chronic infection, suggesting a limited capacity for expansion following PD-1 blockade. To investigate this, CD44^Lo^ and CD44^Hi^ P14 cells were adoptively co-transferred into B6 mice, followed by LCMV clone-13 infection. During the chronic phase, the recipient mice were treated with anti-PD-L1 antibodies, and we analyzed how P14 cells derived from these populations respond to PD-1 blockade (**Figure 5A**). Anti-PD-L1 treatment led to a 1.68-fold increase in CD44^Lo^-derived P14 cells, whereas the CD44^Hi^-derived population showed only a 1.02-fold increase, which was significantly lower than that of the CD44^Lo^ group (**Figure 5B**, **Supplementary Figures 7A, B**). Taken together, these results indicate that CD8 T cells derived from VM cells exhibit limited expansion in response to PD-1 blockade.

## 4 Discussion

In this study, we demonstrated that VM CD8 T cells exhibit poor adaptability under conditions of persistent antigen-stimulation as evidenced by their markedly reduced progeny during the late phase of chronic infection compared to naïve counterparts. Previous studies revealed the importance of TCF1^+^TIM3^–^ stem-like CD8 T cells in maintaining antigen-specific CD8 T cell pools during chronic infection (15–19). Notably, Im and colleagues demonstrated that P14 CD8 T cells lacking the *Tcf7* gene, which encodes TCF1, undergo a substantial loss of the total antigen-specific CD8 T cells, including TIM3^+^ subsets, during the late phase of chronic LCMV infection (18). Similarly, in our study, we observed a marked reduction of virus-specific CD8 T cells derived from VM CD8 T cells at the late phase of chronic LCMV infection. This loss is likely driven by impaired formation of TCF1^+^TIM3^–^ stem-like cells from VM CD8 T cells during the early phase of infection. These findings could have important implications for understanding CD8 T cell responses, particularly in aged individuals, as prior studies have reported a dramatic increase in VM CD8 T cells concomitant with a decline in naïve CD8 T cells in aged mice (24, 27, 28). Given that cancer incidence increases sharply with advancing age (29, 30), the limited adaptability of VM CD8 T cells to chronic antigen-stimulation observed in the present study may contribute to this increased cancer incidence. Accordingly, examining how naïve and VM CD8 T cells in aged hosts respond to persistent antigen-stimulation may provide important insights into age-associated cancer susceptibility.

Here, we observed that VM CD8 T cells preferentially differentiated into KLRG1^+^PD-1^–^ cells rather than PD-1^+^ CD8 T cells during chronic infection, indicating impaired engagement of the T cell exhaustion program. TOX functions as a master regulator of T cell exhaustion by orchestrating transcriptional and epigenetic programs that sustain exhausted T cells during chronic infection and cancer (31), and its expression is driven by NFAT signaling, activated by chronic TCR stimulation (21, 32). In addition, disruption of the NFAT–TOX axis promotes KLRG1 upregulation and PD-1 downregulation (21). Our data showed that TOX expression was significantly lower in VM-derived CD8 T cells compared to naïve-derived cells following LCMV clone-13 infection. Therefore, it is suggested that lower TOX expression is linked to the compromised T cell exhaustion program and differentiation into the unique KLRG1^+^PD-1^–^ subset in the VM-derived population. In this study, we analyzed the differentiation of naïve and VM CD8 T cells after LCMV clone-13 infection within the same environment using adoptive co-transfer experiments. Thus, this difference of TOX expression is regulated by cell intrinsic factors and defining the molecular mechanisms governing TOX regulation in VM CD8 T cells under persistent antigen-stimulation will therefore be an important topic for future investigation.

VM CD8 T cells share many characteristics with true memory CD8 T cells including phenotype and function (1–3). Unlike naïve CD8 T cells, VM cells display a memory-like phenotype and transcriptional profile as confirmed by expression and transcriptomic analyses (2). Functionally, they can respond more rapidly to acute stimulation and confer greater protection against acute infections than naïve cells (10). In this study, we examined VM CD8 T cell responses during LCMV clone-13 infection and found that they exhibit limited capacity to adapt to chronic antigen-stimulation. Interestingly, a previous study reported that, unlike naïve CD8 T cells, true memory CD8 T cells show markedly diminished responses during chronic infection (33). Together, these findings highlight a previously underappreciated similarity between VM and true memory CD8 T cells in their responses to chronic antigen exposure. Although the ability of true memory CD8 T cells to generate the TCF1^+^ stem-like subset during chronic infection was not addressed in the earlier study, as it predated the discovery of this subset, the differentiation of true memory CD8 T cells is likely governed by mechanisms similar to those regulating VM CD8 T cell differentiation.

PD-1 blockade therapy has demonstrated substantial clinical efficacy, leading to tumor regression across various tumor types. Nevertheless, more than half of patients either fail to respond to PD-1 inhibition or do not achieve durable therapeutic benefit (34–39). Consequently, elucidating the underlying mechanisms of resistance to PD-1 targeted immunotherapy has become an area of intense investigation. In this study, we demonstrated that VM-derived CD8 T cells exhibit limited expansion in response to anti-PD-L1 antibody treatment, likely due to lower TCF1^+^ stem-like cells. These findings suggest that understanding how VM CD8 T cells influence the efficacy of PD-1 blockade could provide important insights into the mechanisms of therapeutic resistance. However, addressing this question is still challenging due to limited information of human VM CD8 T cells. Despite several studies reporting phenotypic markers for VM CD8 T cells in humans as well as their responses to cytokine stimulation (2, 27, 40, 41), antigen-specific VM CD8 T cells and their responses to infections and cancer have not been well characterized in humans. Addressing this knowledge gap could provide novel mechanistic insights into PD-1 therapy resistance and inform the development of more effective immunotherapeutic interventions.

In conclusion, our study demonstrates that VM CD8 T cells exhibit diminished responsiveness to persistent antigen exposure compared to naïve CD8 T cells. Mechanistically, VM CD8 T cells fail to fully engage the T cell exhaustion program due to reduced TOX expression, resulting in a smaller pool of TCF1^+^ stem-like cells and preferential differentiation into the effector-like population. Moreover, the VM-derived population display inferior expansion following PD-1 blockade. These findings uncover an unrecognized functional property of VM CD8 T cells and offer important mechanistic insights into the regulation of antigen-specific CD8 T cell responses during chronic infection and cancer.

## Data Availability

The source data used to generate all graphs are included in the article as tables, and the other raw data supporting the conclusions of this article will be made available by the authors, without undue reservation.
