# Supplementary material for: Limited adaptability of virtual memory CD8 T cells to chronic viral infection
Source: Front Immunol. 2026 May 15;17:1804320. doi: 10.3389/fimmu.2026.1804320 (PMC13237571; doi:10.3389/fimmu.2026.1804320)
Supplement: Supplementary file 1 [file DataSheet1.pdf]

Supplementary Material

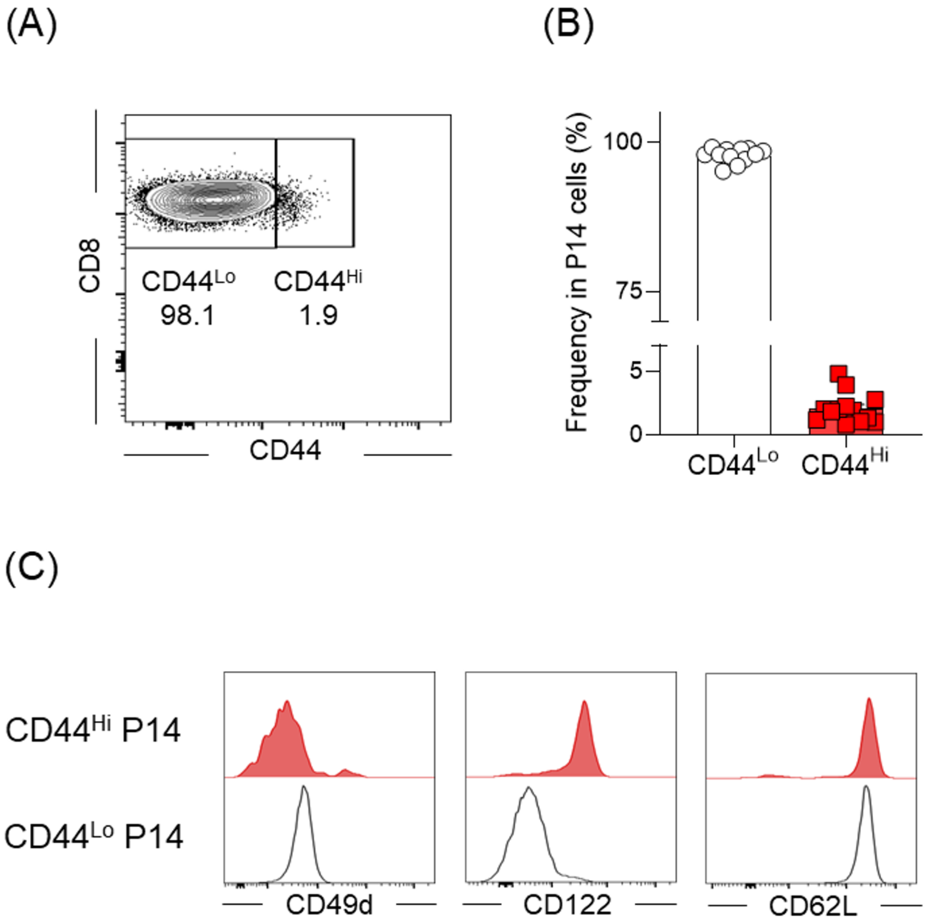

Supplementary Figure 1. Characterization of the phenotype of CD44^{Hi} P14 CD8 T cells. (A) Representative flow plots were gated on P14 CD8 T cells (DbGP33$^+$CD8$^+$ cells). (B) Frequencies of CD44^{Lo} and CD44^{Hi} cells in P14 CD8 T cells. Data are pooled from 5 independent experiments with 13 mice per group. (C) CD49d, CD122, and CD62L expression on CD44^{Lo} and CD44^{Hi} P14 CD8 T cells in the spleen. Representative histograms were gated on either CD44^{Lo} or CD44^{Hi} P14 CD8 T cells.

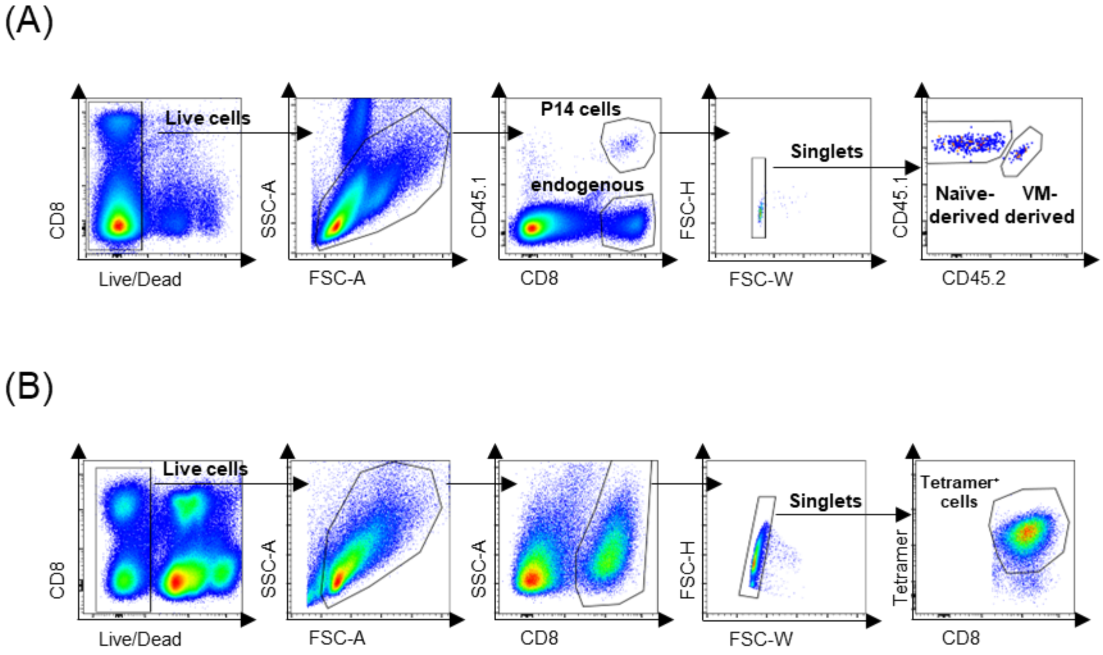

Supplementary Figure 2. Gating strategies for flow cytometric analyses. (A) Gating strategy for experiments analyzing CD44^{Lo}- and CD44^{Hi}-derived P14 CD8 T cell responses during LCMV infection. After gating on live lymphocytes, donor P14 (CD45.1^{+}CD8^{+}) and endogenous (CD45.1^{-} CD8^{+}) CD8 T cells were identified based on the expression of CD45.1, a congenic marker. Doublets were excluded from the P14 CD8 T cell population, and naïve-derived (CD45.1$^{+/+}$) and VM-derived (CD45.1^{+}/CD45.2^{+}) P14 populations were distinguished by CD45.2 expression. As shown in Figures 2–4, the phenotypes of naïve- and VM-derived populations were subsequently analyzed. The endogenous CD8 T cell population was used to determine gates for PD-1, KLRG1, granzyme B, and TOX expression, as this population includes naïve CD8 T cells that lack PD-1, KLRG1, granzyme B, and TOX expression. (B) Gating strategy for cells following tetramer enrichment in experiments analyzing the responses of antigen-specific non-transgenic polyclonal CD44^{Lo} and CD44^{Hi} CD8 T cells during LCMV clone-13 infection. After gating on live CD8 T cells, doublets were excluded and tetramer^{+} (DbGP33^{+} and DbGP276^{+}) cells were then identified. As shown in Figures 1F and 1G, frequencies and absolute cell numbers of donor-derived tetramer^{+} cells were subsequently analyzed.

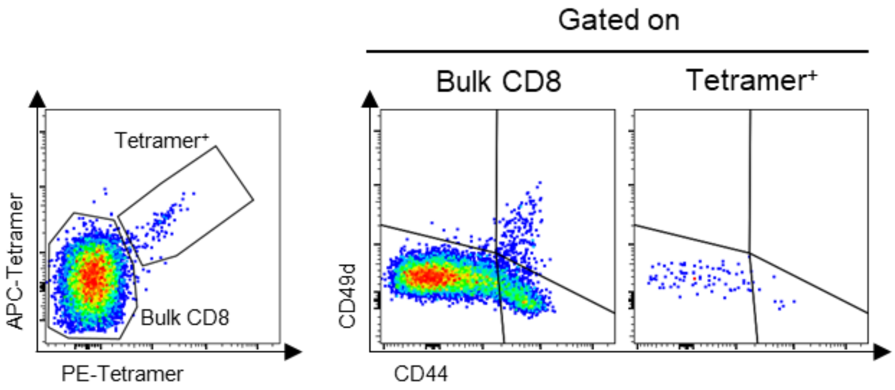

Supplementary Figure 3. The phenotype of LCMV-specific non-transgenic polyclonal CD8 T cells obtained from uninfected B6 mice. The spleen was harvested from LCMV uninfected B6 mice and tetramer enrichment was performed to examine the phenotype of DbGP33^{+} and DbGP276^{+} endogenous CD8 T cells. Representative plots show CD44 and CD49d expression in tetramer^{+} CD8 T cells. Bulk tetramer^{-} CD8 T cells were used to determine the gate for CD44 and CD49d expression, as this population includes naïve (CD44^{Lo}), VM (CD44^{Hi}CD49d^{Lo}), and true memory (CD44^{Hi}CD49d^{Hi}) CD8 T cells.

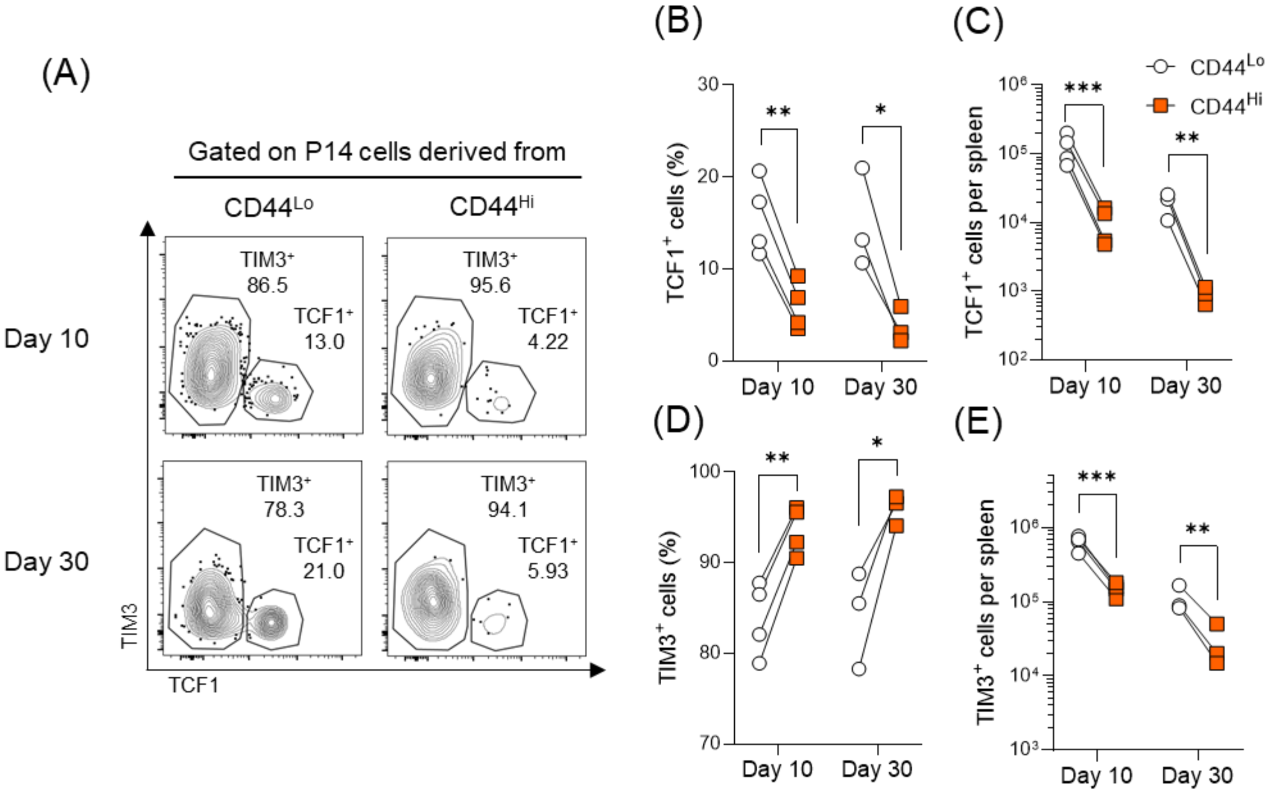

Supplementary Figure 4. Impaired formation of TCF1$^+$ stem-like cells in the presence of CD4 T cells during LCMV clone-13 infection. Congenically marked CD44^{Lo} and CD44^{Hi} P14 CD8 T cells (1,000 cells each) were adoptively co-transferred into B6 mice, followed by LCMV clone-13 infection. These mice were not treated with the anti-CD4 antibody. (A) Representative flow plots were gated on P14 CD8 T cells derived from either CD44^{Lo} or CD44^{Hi} cells on days 10 and 30 post-LCMV clone-13 infection. Gating strategy is shown in Supplementary Figure 2A. (B–E) Percentages of TCF1$^+$ and TIM3$^+$ progeny derived from CD44^{Lo} and CD44^{Hi} P14 CD8 T cells (B and D) and their absolute numbers (C and E) in the spleens on days 10 and 30 post-LCMV clone-13 infection. Data are representative of 2 independent experiments with 3 or more mice per group. Each symbol represents an individual mouse, and lines indicate paired comparisons within the same mice. Statistical analyses were performed using multiple paired *t* test. * $p < 0.05$; ** $p < 0.01$; *** $p < 0.001$.

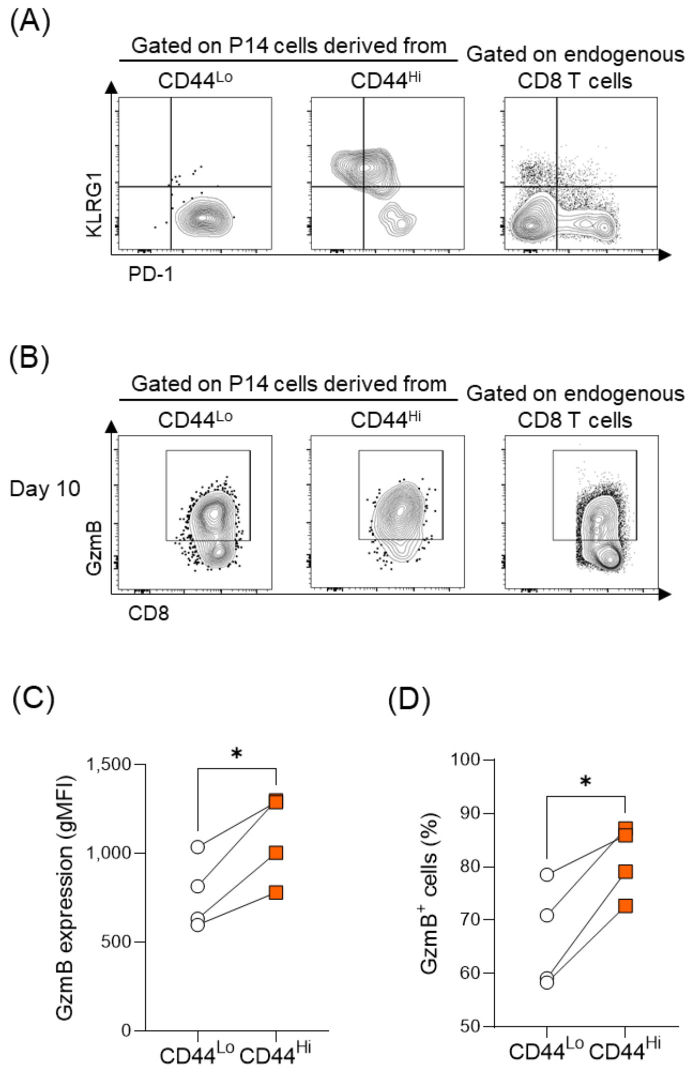

Supplementary Figure 5. PD-1, KLRG1, and granzyme B expression in naïve- and VM-derived P14 CD8 T cells during chronic infection. Experimental design is shown in Figure 1A. Gating strategy is shown in Supplementary Figure 2A. (A) Representative flow plots were gated on P14 CD8 T cells derived from either $CD44^{Lo}$ or $CD44^{Hi}$ cells, or endogenous CD8 T cells on day 34 post-LCMV clone-13 infection. The gate for PD-1 and KLRG1 expression was determined using the plot from endogenous CD8 T cells containing naïve CD8 T cells that lack PD-1 and KLRG1 expression. (B) Representative flow plots were gated on P14 CD8 T cells derived from either $CD44^{Lo}$ or $CD44^{Hi}$ cells, or endogenous CD8 T cells on day 10 post-LCMV clone-13 infection. The gate for granzyme B (GzmB) expression was determined using the plot from endogenous CD8 T cells containing naïve CD8 T cells that lack GzmB expression. (C and D) Graphs show GzmB expression (C: gMFI) and the frequency of $GzmB^+$ cells (D) in progeny derived from $CD44^{Lo}$ and $CD44^{Hi}$ P14 CD8 T cells in the spleens on day 10 post-LCMV clone-13 infection. Data are representative of 2 independent experiments with 4 mice per group. Each symbol represents an individual mouse, and lines indicate paired comparisons within the same mice. Statistical analyses were performed using paired *t* test. * $p < 0.05$.

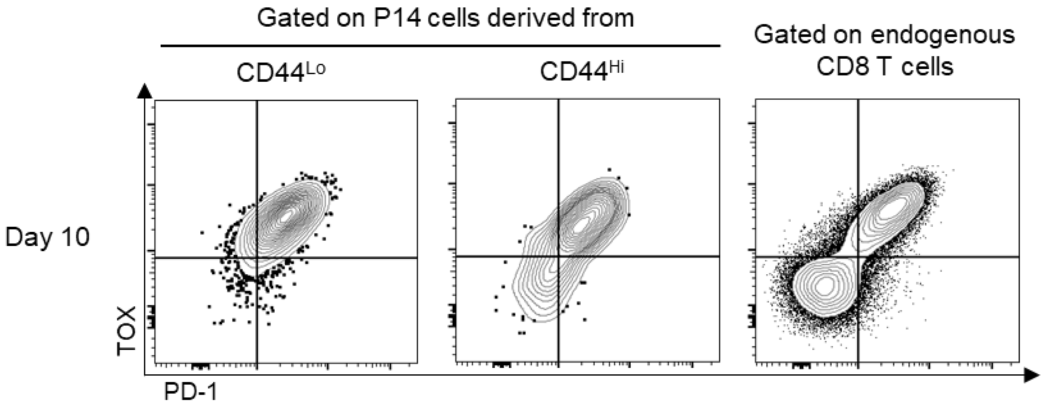

Supplementary Figure 6. PD-1 and TOX expression in naïve- and VM-derived P14 CD8 T cells during chronic infection. Experimental design is shown in Figure 1A. Gating strategy is shown in Supplementary Figure 2A. Representative flow plots were gated on P14 CD8 T cells derived from either $CD44^{Lo}$ or $CD44^{Hi}$ cells, or endogenous CD8 T cells on day 10 post-LCMV clone-13 infection. The gate for PD-1 and TOX expression was determined using the plot from endogenous CD8 T cells containing naïve CD8 T cells that lack PD-1 and TOX expression.

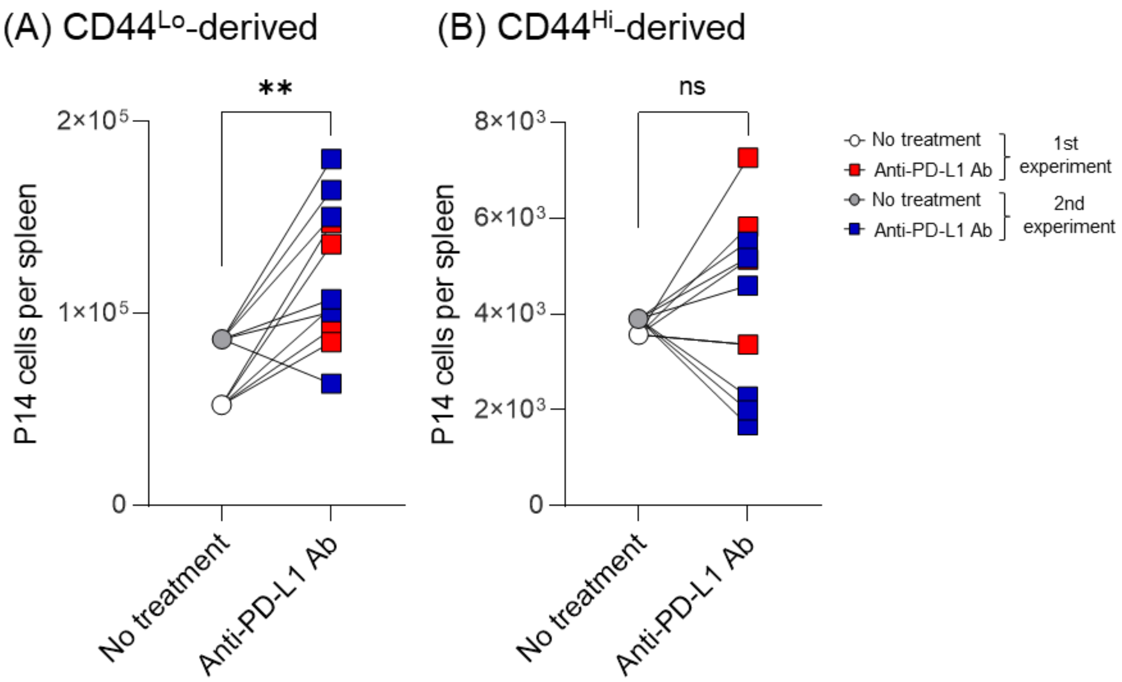

Supplementary Figure 7. VM-derived CD8 T cells exhibit limited expansion following PD-1 blockade. Experimental design is shown in Figure 5A. (A and B) Graphs show absolute numbers of $CD44^{Lo}$-derived (A) and $CD44^{Hi}$-derived (B) P14 CD8 T cells per spleen after anti-PD-L1 antibody treatment. The absolute cell numbers were compared to the geometric mean of absolute P14 numbers in untreated group from each experiment (1st experiment: $n = 4$; 2nd experiment: $n = 5$). Data are pooled from 2 independent experiments and each symbol in the anti-PD-L1 antibody treated group represents an individual mouse. Statistical analyses were performed using paired t test. ns, not significant; ** $p < 0.01$.
